# Bell’s palsy during interferon alpha 2a treatment in a case with Behçet uveitis

**DOI:** 10.12688/f1000research.2-245.v1

**Published:** 2013-11-15

**Authors:** Fatime Nilüfer Yalçindağ, Cem Alay

**Affiliations:** 1 Department of Ophthalmology, Ankara University Faculty of Medicine, Ankara, Turkey

## Abstract

**Purpose: **To present a case who developed Bell’s palsy while using interferon alpha 2a for Behçet uveitis.

**Methods: **A patient with Behçet disease presented with decreased vision in his right eye. Ophthalmic examination, fundus fluorescein angiography and optical coherence tomography were performed. After developing facial paralysis while on interferon therapy, the patient was referred to our neurology service for differential diagnosis and treatment.

**Results: **Examination of right eye revealed panuveitis with branch retinal vein occlusion, so high dose steroids were prescribed. In three days there was no improvement in terms of vitreous inflammation and so steroids were replaced with interferon. At the seventh month, patient experienced a facial paralysis. After eliminating other causes, including viral infections, trauma, cold exposure and neurological evaluation with cranial MRI, the patient was diagnosed to have Bell’s palsy by a neurologist. Interferon was replaced with mycophenolate mofetil and the Bell’s palsy was treated with oral steroids.

**Conclusion: **It is important to be alert to both common and rare complications while treating with interferon.

## Introduction

Interferon alpha 2a is a recombinant biological agent that is used for the treatment of several diseases including chronic hepatitis C, hairy cell leukemia, Philadelphia chromosome positive chronic myelogenous leukemia (CML) and AIDS-related Kaposi’s sarcoma. It is also used in Behçet uveitis that is refractory to conventional immunosuppressive agents ^1, 2^. The central (CNS) and peripheral nervous system (PNS) can be affected while using interferon alpha 2a. There are two existing case reports describing Bell’s palsy associated with interferon treatment for chronic hepatitis C infection ^3, 4^. Here we present another case report indicating Bell’s palsy during interferon alpha 2a treatment in Behçet uveitis.

## Case report

A 27-year old Turkish, male, taxi driver who has Behçet’s Disease was referred to our clinic for a decrease in visual acuity in his right eye for 2 days. At presentation, the best-corrected visual acuity was 20/320 in his right eye and 20/20 in his left eye. On slit lamp examination, there were keratic precipitates, anterior chamber cells and vitreous cells in his right eye. Fundus examination revealed optic disc edema, macular edema and abundant intraretinal hemorrhages in the inferior temporal part of retina. His left eye seemed to be normal. Optical coherence tomography (Cirrus HD-OCT Model 4000, Carl Zeiss Meditec) (OCT) and fluorescein angiography (FA) was then performed. Central macular thickness (CMT) was 595 µm on OCT. In FA, there was mild hyperfluorescence on both optic discs. In addition, hypofluorescence in the inferior retinal areas due to hemorrhages and staining on the vessel walls in right eye was spotted. Macular edema and peripheral ischemia were also evident. A diagnosis of panuveitis with inferior temporal branch retinal vein occlusion due to Behçet’s Disease was made.

Firstly, high dose steroids (1 gram/day methylprednisolone, intravenously) were prescribed to the patient for 3 days. Dexamethasone eye drops every hour during the day and topical cyclopentolate 1% three times a day were also prescribed. On the third day, the anterior chamber reaction seemed to be decreased but no improvement was observed, either in the vitreous inflammation or the macular edema. As such, the initial high dose steroid treatment was stopped and interferon alpha 2a (Roferon-A, Roche Pharmaceuticals, Hoffmann-La Roche Inc., Switzerland) 4.5 million IU (MIU) subcutaneously on alternate days and 10 mg/day oral prednisolone were prescribed to the patient. At that point, we had decided upon interferon treatment because the patient was young and the first ocular presentation of Behçet’s disease was seriously threatening in terms of visual prognosis. In addition, green argon laser photocoagulation was applied to the ischemic areas of the retina.

The patient was examined weekly for the first month in order to observe the effects of treatment closely. After this, the follow-up was done monthly. In the first month, steroid treatment was tapered slowly and stopped while interferon treatment was continued. In the first 6 months, no adverse effects were observed, best corrected visual acuity improved to 20/32 and the panuveitis and macular edema regressed. CMT was 230 µm after the 6-months of interferon alpha 2a therapy. There were no signs of anterior uveitis or vitreous inflammation. The patient’s oral aphthae, genital ulcers and arthralgia (symptoms of Behçet’s Disease) were also improved. At this stage, the dose of interferon alpha 2a was tapered to 3 MU on alternate days.

One month after tapering the dose, the patient experienced difficulty in closing his right eyelid and stiffness in the right half of his face. His wife also reported that there had been no movement on the right side of his face for about 15 days. There was no history of trauma, infection, cold exposure or any other predisposing factors that might have explained this situation. The patient was tested for several viral infections including herpes viruses, cytomegalovirus (CMV) and Epstein-Barr virus (EBV). All tests were negative. Afterwards, in order to eliminate neurological problems such as brain tumor, stroke or neuro-Behçet disease, the patient was immediately referred to our neurology service. Cranial MRI was normal and the neurological evaluation revealed no pathological findings apart from facial paralysis. As such the patient was diagnosed with Bell’s palsy. Interferon use was considered as a possible reason for this condition and so interferon treatment was tapered and discontinued. Mycophenolate mofetil (720 mg/day, oral) was prescribed for Behçet’s disease because the patient had previously experienced nausea and vomiting with azathioprine, which had been prescribed before as an initial therapy for Behcet’s disease by a rheumatologist. Oral methylprednisolone 48 mg per day was prescribed by the neurologist and gradually tapered (8 mg per week) over 6 weeks. In addition, a program of exercises for the facial muscles was recommended. All symptoms of Bell’s palsy disappeared within 2 months of onset.

## Discussion

There is a wide variety of adverse effects due to the use of interferon alpha 2a, including flu-like syndrome (unusual tiredness, fever, chills, muscle aches, and joint pain), injection site reaction (redness, pain at the site of injection), proneness to serious infections, new or worsening autoimmune disease ^5– 7^, myelosuppression ^8, 9^, depression, suicide, suicidal thoughts, and cardiovascular problems ^10, 11^ such as hypotension or hypertension, arrhythmia and myocardial infarction. Patients may also suffer from nausea, vomiting, loss of appetite, diarrhea, cough, dry mouth, dizziness, vertigo, sweating, itching, hair loss, fatigue, abdominal pain, constipation, sore throat, insomnia ^12^, anxiety and numbness.

Interferon alpha 2a also affects the CNS and PNS. Difficulty of concentration and aggression are the most common CNS problems. Decrease in mental activity, dysphasia, dysarthria, aphasia, aphonia, amnesia, multiple sclerosis-like disease ^13^, transient ischemic attacks, encephalopathy, confusion, somnolence and coma may also occur during the use of interferon. It is also known that several symptoms such as ataxia, paresthesia, tremor and peripheral neuropathies ^14– 18^ can be seen if the PNS is affected. In our patient, we encountered Bell’s palsy, a peripheral neuropathy affecting the facial nerve in the 7 ^th^ month of interferon therapy. No elucidating factors but interferon were present at the time we diagnosed the patient. Hitherto, there have been two other reports of Bell’s palsy related to the use of interferon ^3, 4^. So it was important for us to call attention to our case as an example of a rare complication of interferon use. In conclusion, physicians should always keep an eye on patients who are being treated with interferon alpha 2a. Physicians should be alert to the rare side effects of interferon treatment as well as the more common and well-known side effects of this agent.

## Consent

Written informed consent for publication of his clinical details was obtained from the patient.

## References

[1] Kötter I Zierhut M Eckstein AK: Human recombinant interferon alfa-2a for the treatment of Behçet's disease with sight threatening posterior or panuveitis. *Br J Ophthalmol.* 2003;87(4):423–31 10.1136/bjo.87.4.423 12642304 PMC1771623

[2] Yalçindağ FN Uzun A: Results of Interferon Alpha-2a Therapy in Patients with Behcet's Disease. *J Ocul Pharmacol Ther.* 2012;28(4):439–43 10.1089/jop.2011.0238 22455657

[3] Ogundipe O Smith M: Bell’s palsy during interferon therapy for chronic hepatitis C infection in patients with haemorrhagic disorders. *Haemophilia.* 2000;6(2):110–2 10.1046/j.1365-2516.2000.00391.x 10781198

[4] Hoare M Woodall T Alexander GJ: Bell’s palsy associated with IFN-alpha and ribavirin therapy for hepatitis C virus infection. *J Interferon Cytokine Res.* 2005;25(3):174–6 10.1089/jir.2005.25.174 15767792

[5] Rizvi R Hojjati M: Interferon-α induced lupus in a patient with chronic hepatitis C virus. *J Clin Rheumatol.* 2011;17(3):152–3 10.1097/RHU.0b013e31821557e7 21441813

[6] Savvas SP Papakostas N Giannaris M: Interferon alpha-induced hashimoto thyroiditis followed by transient graves disease in a patient with chronic HCV infection. *South Med J.* 2010;103(6):585–8 10.1097/SMJ.0b013e3181ddd952 20710148

[7] Yang D Arkfeld D Fong TL: Development of anti-CCP-positive rheumatoid arthritis following pegylated interferon-α2a treatment for chronic hepatitis C infection. *J Rheumatol.* 2010;37(8):1777 10.3899/jrheum.100092 20675854

[8] Zizer E Bommer M Barth T: Severe agranulocytosis as a rare side effect of pegylated interferon therapy for chronic hepatitis B. *Z Gastroenterol.* 2011;49(5):596–8 10.1055/s-0029-1245768 21557170

[9] Hajder J Stanisavljević N Marković O: Late onset of severe thrombocytopenia during interferon treatment for chronic hepatitis C infection--case report. *Srp Arh Celok Lek.* 2010;138(3–4):240–3 10.2298/SARH1004240H 20499509

[10] Roche NC Paule P Kerebel S: Complete atrio-ventricular block: a rare complication of interferon alpha therapy. *Presse Med.* 2011;40(3):316–8 10.1016/j.lpm.2010.10.024 21242049

[11] Velasco J Oriñuela I Sanjuán AZ: Pericardial effusion associated to interferon in an immunocompetent patient. *Enferm Infecc Microbiol Clin.* 2010;28(10):749–50 10.1016/j.eimc.2010.02.019 20579776

[12] Raison CL Rye DB Woolwine BJ: Chronic interferon-alpha administration disrupts sleep continuity and depth in patients with hepatitis C: association with fatigue, motor slowing, and increased evening cortisol. *Biol Psychiatry.* 2010;68(10):942–9 10.1016/j.biopsych.2010.04.019 20537611 PMC2937202

[13] Matsuo T Takabatake R: Multiple sclerosis-like disease secondary to alpha interferon. *Ocul Immunol Inflamm.* 2002;10(4):299–304 10.1076/ocii.10.4.299.15588 12854039

[14] Knyazer B Lifshitz T Marcus M: Anterior ischemic optic neuropathy in a patient with hepatitis C treated with interferon-alpha and ribavirin. *Isr Med Assoc J.* 2011;13(4):251–3 21598818

[15] Khiani V Kelly T Shibli A: Acute inflammatory demyelinating polyneuropathy associated with pegylated interferon alpha 2a therapy for chronic hepatitis C virus infection. *World J Gastroenterol.* 2008;14(2):318–21 10.3748/wjg.14.318 18186575 PMC2675134

[16] Tunca A Erbayrak M Aytaç S: Axonal neuropathy and hearing loss associated with alpha interferon treatment in chronic hepatitis B: a case report. *Turk J Gastroenterol.* 2004;15(2):97–9 15334319

[17] Fukumoto Y Shigemitsu T Kajii N: Abducent nerve paralysis during interferon alpha-2a therapy in a case of chronic active hepatitis C. *Intern Med.* 1994;33(10):637–40 10.2169/internalmedicine.33.637 7827383

[18] Bauherz G Soeur M Lustman F: Oculomotor nerve paralysis induced by alpha II-interferon. *Acta Neurol Belg.* 1990;90(2):111–4 2114727

