# Supplementary material for: Bell’s palsy during interferon alpha 2a treatment in a case with Behçet uveitis
Source: F1000Res. 2013 Nov 15;2:245. [Version 1] doi: 10.12688/f1000research.2-245.v1 (PMC3962000; doi:10.12688/f1000research.2-245.v1)
Supplement: Peer review history

# Peer review: Referee response for version 1

**Published:** 2014-02-10

**Authors:** Sumru Onal

**Affiliations:** 1 Uveitis Service, Department of Ophthalmology, Marmara University School of Medicine, Uskudar, Istanbul, Turkey

This manuscript by Yalcindag and Alay reports on a patient with Bell’s palsy that occurred as a possible adverse effect of interferon alfa-2a that was prescribed in an effort to control Behçet uveitis. It is a well-written paper and will contribute to practicing ophthalmologists. My comments are listed below:

 

1. I would suggest that the authors name the disease as “Behçet disease” rather than “Behçet’s disease” throughout the paper. The intraocular inflammation has already been well addressed as “Behçet uveitis”.
   
2. There is need for revision of the English grammar and language.
   
3. I would recommend that the authors include information from the World Health Organization’s causality assessment of suspected adverse drug reactions in the discussion section. The authors should also classify their observations based on this classification. It seems to me that the described finding is a possible adverse effect. The reference would be: “Edwards IR, Biriell C *.* Harmonisation in pharmacovigilance *. Drug Saf *1994;10:93–102.” The classification is as follows:
**Certain: **A clinical event, including laboratory test abnormality, occurring in a plausible time relationship to drug administration that cannot be explained by concurrent disease or other drugs or chemicals. The response to withdrawal of the drug (dechallenge) should be clinically plausible. The event must be definitive pharmacologically or phenomenologically, using satisfactory rechallenge procedure if necessary.
**Probable/likely: **A clinical event, including laboratory test abnormality, with a reasonable time sequence to administration of the drug, unlikely to be attributed to concurrent disease or other drugs or chemicals, which follows a clinically reasonable response on withdrawal (dechallenge). Rechallenge information is not required to fulfil this definition.
**Possible: **A clinical event, including laboratory test abnormality, with a reasonable time sequence to administration of the drug, which could also be explained by concurrent disease, or other drugs or chemicals, or underlying disease provide plausible explanations.
**Unlikely: **A clinical event, including laboratory test abnormality, with a temporal relationship to drug administration that makes a causal relationship improbable and in which other drugs, chemicals, or underlying disease provide plausible explanations.
**Conditional/unclassified: **A clinical event, including laboratory test abnormality, reported as an adverse reaction, about which more data are essential for a proper assessment or the additional data are undergoing examination.
**Nonassessable/unclassified: **A report suggesting an adverse reaction that cannot be judged because information is insufficient or contradictory and that cannot be supplemented or verified.

I have read this submission. I believe that I have an appropriate level of expertise to confirm that it is of an acceptable scientific standard.

# Peer review: Referee response for version 1

**Published:** 2014-01-14

**Authors:** Erkan Alpsoy

**Affiliations:** 1 Department of Dermatology and Venerology, Faculty of Medicine, Akdeniz University, Antalya, Turkey

This is a well-organised and well-written paper and I am sure that it will be helpful to readers in this field. I only have one minor comment: that the English Language should be checked and grammatical mistakes corrected.

I have read this submission. I believe that I have an appropriate level of expertise to confirm that it is of an acceptable scientific standard.
